# Sensors: future tools for detecting young patient’s stress during a dental invasive versus a non-invasive dental treatment—a pilot study

**DOI:** 10.1007/s40368-024-00967-7

**Published:** 2024-11-14

**Authors:** C. Jaldin, C. Jonasson, T. Fagrell, A. Robertson, L. Krekmanova

**Affiliations:** 1 Public Dental Service, Region Vastra Gotaland, Gothenburg, Sweden; 2 RISE Research Institutes of Sweden, Gothenburg, Sweden; 3 Institute of Odontology, Sahlgrenska Academy, University of Gothenburg, Gothenburg, Sweden; 4 Department of Pediatric Dentistry, Institute of Odontology, Sahlgrenska Academy, Gothenburg, Sweden

**Keywords:** Children, Adolescents, Dentistry, Innovation, Stress, Pain, Fear, Prevention

## Abstract

**Aim:**

A reliable tool to visualise children’s early stress signs to prevent dental fear development is needed. The aim was to evaluate the commercially available, CE marked, Shimmer3 GSR + unit’s ability to indicate for stress as a reaction of fear or pain for a non-invasive dental treatment (NI) and an invasive dental treatment (I).

**Methods:**

Patients 14–16 years old were invited to undergo an oral check-up (NI) or an orthodontic premolar extraction (I), respectively. Digital data, measured via electrodes and optical pulse probe, placed on the wrist and fingers, monitored by the Shimmer3 GSR + unit, was transferred via Bluetooth to the HP-laptop. The observed digital parameters were: heart rate based on photoplethysmography (PPG), galvanic skin response (GSR), and 3-axis gyroscope and accelerometer signals for hand movements. Protocols for patient self-report scales were used: coloured analogue scale for pain intensity, facial analogue scale for the mood, and a dental fear scale. Descriptive statistics was performed.

**Results:**

The NI-group: 20 patients, (14.6 ± 0.5 years), underwent 20 oral check-ups. The I-group: 14 patients, (15.3 ± 0.5 years), underwent 28 premolar extractions. All patients tolerated the Shimmer3 GSR + unit well. The GSR signal increased significantly, at start and during the oral injection, in the I-group. The GSR amplitudes persisted throughout and post the dental injection. No general uniform pattern or high GSR amplitudes were produced regarding NI-group.

**Conclusions:**

Considering the limitations of this study, the following conclusions can be made: the invasive treatment resulted in a specific unison GSR pattern, while the non-invasive procedure showed individually scattered GSR reactions. The commercially available CE-marked Shimmer3 GSR + device indicated the patient's stress response triggered by the invasive anaesthetic procedure.

## Introduction

Non-invasive mobile health monitoring systems have been developed and utilised in the field of health promotion and medicine (Mao et al. 2023; Muzny et al. 2020; Liao et al. 2019; Murphy et al. 2019; Lu et al. 2018; Kamišalić et al. 2018). In addition, continuous monitoring and collecting of physiologic al variables via sensors have been tested, to facilitate medical diagnosis and individually tailored treatments (Murphy et al. 2019). A care-related area that would benefit from live monitoring patients’ physiological reactions, via a non-invasive device, is the paediatric dentistry field as: children and adolescents do not always have the ability, courage, or vocabulary to express their negative perceptions during dental procedures (Krekmanova and Robertson 2020). Furthermore, perceived anxiety, fear or pain may eventually lead to consequences, such as behavioural problems, dental fear, and non-attendance from subsequent dental care appointments (Klingberg and Broberg 2007; Ghanei et al. 2018). In addition, young dental patients with a shy, insecure or introvert temperament may need additional support to communicate their own needs. The inability of these young individuals to stand up for themselves is exemplified in a 5-year prospective study of 3–19-year-olds, a majority of whom cooperated with invasive dental procedures, despite experiencing fear and pain (Krekmanova and Robertson 2020; Ghanei et al. 2018). In the same study, patients reported the dental check-ups i.e. non-invasive treatment, to provoke significantly lower levels of fear and pain, than invasive procedures (Krekmanova and Robertson 2020; Ghanei et al. 2018). The results highlighted patient’s exposure in the dental setting and needless suffering during procedures such as oral injections and dental extractions.

Seen from the dental care perspective, unexperienced dental staff's inadequate sensitivity to patient’s subtle reactions may increase a negatively perceived situation (Krekmanova et al. 2021). The above reasoning identifies an area in need of development. A desirable solution would be to visualise young patient’s reactions and so increase the dental staff’s sensitiveness, preferably by reading the patient’s physiological parameters during dental treatments. Thus, the physiological response would not depend on each patient’s ability to communicate, which broadens the clinical implementation. However, a clinical requirement would be to use a device that is easily manageable by dentists and effortlessly accepted among patients.

Among commercially available devices on the market, the CE-marked Shimmer3 GSR + unit measures galvanic skin response (GSR), heart rate (HR) and hand movements, therefore, considered eligible to be tested for its ability to record physiological changes in the dental patient (https://shimmersensing.com).

## Aim

The aim was to evaluate the commercially available, CE marked, Shimmer3 GSR + unit’s ability to indicate for stress as a reaction of fear, regarding a non-invasive dental treatment (NI), and for an invasive dental treatment (I).

## Methods

### Study design

The study design was experimental, aiming to evaluate the Shimmer3 GSR + unit's ability to indicate the patient’s negative reactions during different dental interventions.

### Location of the study and patients

The pilot study was conducted at the Public Dental Service. In addition, the survey was performed in collaboration between different cooperation partners (data blinded).

### Inclusion criteria

*Patient listed at the specific Dental Public Clinic for the study performance.
*Patient 14–16 years old, with no medical diagnoses or medications.
*Patient and legal guardian fluent in the blinded language as each patient should be introduced to the validated scales: coloured analogue scale (CAS); 0 = no pain to 10 = most possible pain, for pain intensity, facial analogue scale (FAS); A = happy to I = crying, for the emotional state, and a fear scale; 0–4, 0 = not afraid to 4 = terrified, to enable to communicate the own experience.
*An impending dental check-up.
*An impending dental extraction of a permanent premolar, due to orthodontic indications.

### Exclusion criteria

*Patient in need of sedatives or nitrous oxide sedation.

### Intervention groups

- Non-invasive treatment group (NI); an oral check-up.
- Invasive treatment group (I); a premolar extraction, maxilla, or mandible due to orthodontic indication.

### Study information and group allocation

Patients with impending oral examinations/extractions who met the inclusion criteria were assigned to either group NI or group I. Patients were then introduced to the purpose of the study, and the anonymous data processing, both verbally and through written information. Written and verbal informed consent from the patient and guardian was required for study eligibility.

Voluntariness was emphasised, highlighting that the participation could be withdrawn at any time during the study.

No compensation was given to the informants.

### Sample size

The sample size in this pilot study presumed that the participant number of 20 patients, in NI and I-group, would be sufficient to display differences between the groups. Based on clinical studies, it was anticipated that the I-group would show different physiological reactions compared to the NI-group.

### Shimmer GSR + unit

**Fig. 1:**
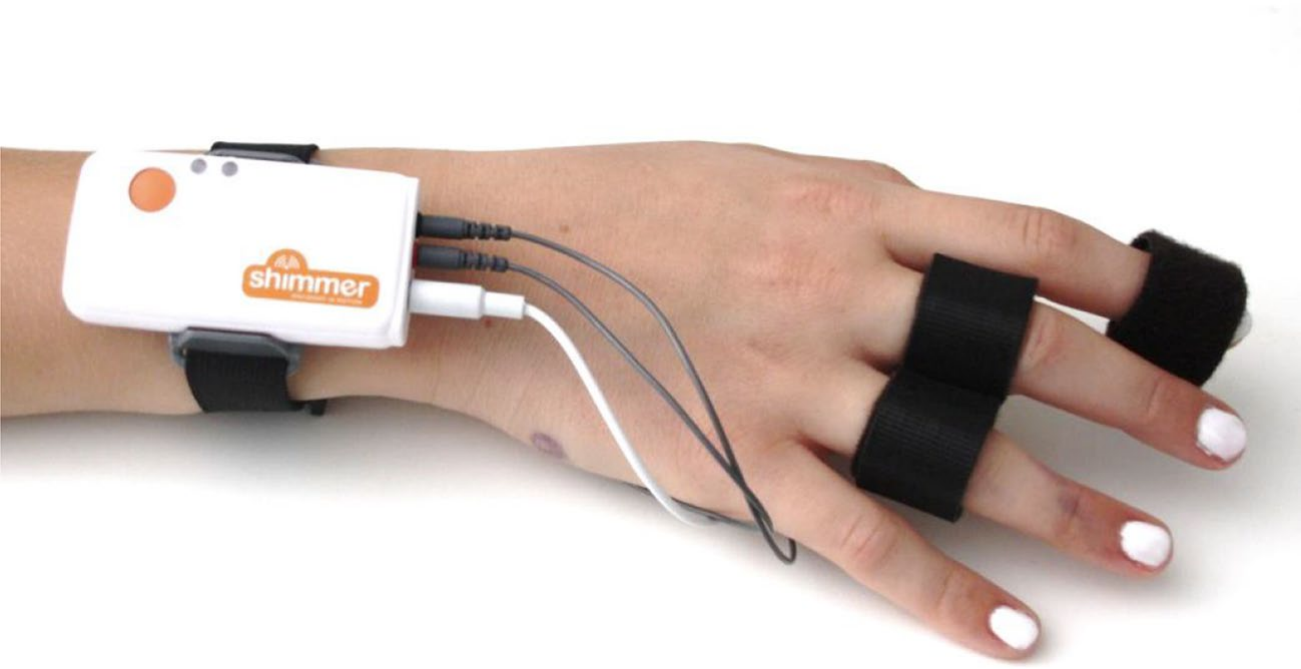
Shimmer3 GSR + unit, a wireless sensor device monitoring skin conductivity, heart rate and hand movements

Shimmer3 GSR + unit is a wireless sensor device that monitors skin conductivity, heart rate and hand movements (Fig. 1). It provides connections and preamplification for one channel of GSR data acquisition. The GSR + unit measures the skin conductivity between two reusable electrodes attached to two fingers of the hand. Alternatively, the sensor is also compatible with disposable electrodes that can be attached to the palmar surface or any other part of the body. Shimmer3 GSR + also provides an additional channel that can capture the optical pulse or PPG (plethysmograph) signal to estimate the heart rate. It also has built-in motion sensors (accelerometer, gyroscope, and magnetometer) that can be used to capture hand movements and orientation. Designed to be wearable, the GSR + unit is free from wired constraints and provides reliable data via a wireless BLE transmitter. Reusable finger electrodes, disposable adhesive electrodes, and wrist strap are included.

## Data collection

### Clinical setting and dentist calibration

The pilot study was performed January through December 2023. A general dentist and dental assistant team executed all oral check-ups and premolar extractions.

The general dentist (CJa) responsible for conducting the collection of the data was instructed and trained to use the Shimmer3 GSR + unit by author (CJo), and the clinical performance and protocol parameters by author (LK).

#### Analogue and digital data

All analogue data were collected during the NI and I dental appointments, using an intervention protocol for each group, respectively. Patient reactions for each included protocol item were time indicated and documented regarding emotion, pain intensity, and fear. The dentist CJa assessed patient cooperation on a four graded scale: 0 = physical resistance/crying, 1 = reluctant acceptance/mild protests, 2 = reluctant/indifferent acceptance, 3 = full acceptance (Rud and Kissling 1973).

The digital parameters were continuously recorded and electronically stored.

### Patient preparation

Prior to each NI and I treatment, the patient was introduced to the analogue scales regarding expressing anxiety, fear, and pain. The patient was then seated for 5 min before applying the Shimmer3 GSR + unit, to create a calm starting position.

### Non-invasive treatment group

Patient was seated in the dental chair for 5 min. The Shimmer3 GSR + unit bracelet was then applied on the left or right wrist. The GSR electrodes were attached to two fingers, and the optical probe to one finger. The intervention was stepwise proceeded by the dentist CJa guiding each patient in accordance with the protocol:

*Seated patient, *Lying patient, *Dentist fingers into the patient’s mouth, *Dental mirror usage, *Dental probe usage, *Oral radiograph (individual indication/not mandatory) and *Fluoride-varnish appliance (individual indication/not mandatory).

After completion of treatment, the patient gave a self-report regarding CAS, FAS and regarding fear (0–4), for each protocol item. The dentist assessment regarding patient fear and cooperation (0–4) was included in the protocol.

The clock time for each protocol step was observed and recorded by the assigned assistant, providing the opportunity to relate to a corresponding physiological value. Negative reactions of the patients were observed and addressed by CJa. In order to meet national recommendations, interventions were carried out in accordance with the Tell Show Do method (Holst and Ek 1988; Addleston 1959). Correspondingly, CJa would stop and communicate/adjust for the patient’s experience of discomfort.

Patient was seated in the dental chair for 5 min. The Shimmer3 GSR + unit bracelet was then applied on the left or right wrist. The GSR electrodes were attached to two fingers, and the optical probe to one finger. The intervention was stepwise proceeded by the dentist CJa guiding each patient in accordance with the protocol:

*Seated patient, *Lying patient, *Dentist fingers into the patient’s mouth, Dental mirror usage, *Dental probe usage (individual indication), *Topical anaesthesia (5 min) *Local anaesthesia, *Inferior Alveolar Nerve Block, *Trans papillary anaesthesia, *Dental luxation and *Dental extraction.

The administration of local anaesthesia was standardised by making the protocol steps *Topical anaesthesia (5 min), *Local anaesthesia and *Transpapillary anaesthesia mandatory for every extraction procedure.

After completion of treatment, the patient gave a self-report regarding CAS, FAS and regarding fear (0–4), for each protocol item. The dentist assessment regarding patient fear and cooperation (0–4) was included in the protocol.

The clock time for each protocol step was observed and recorded by the assigned assistant, providing the opportunity to relate to a corresponding physiological value. Negative reactions of the patients were observed and addressed by CJa. In order to meet national recommendations, interventions were carried out in accordance with the Tell Show Do method. Correspondingly, CJa would stop and communicate/adjust for the patient’s experience of discomfort.

### Data readings

The stored data readings from the Shimmer3 GSR + device were related to the NI and I protocol-variables, respectively. Each variable was correlated to the patient’s physiological response in time.

After completion of treatment, each patient gave self-report on the CAS, FAS, and regarding fear, for each protocol item, respectively. The dentist assessment regarding patients fear and cooperation (0–3): 0 = no cooperation to 3 = full cooperation, was included in the protocol.

Analogue patient data were collected in binders, while digital data were stored on a USB flash drive. All data were saved in a locked space. During the clinical study implementation, only the author CJa, LK had access to the locked space. All data were compiled and processed by CJa, CJo, and LK.

### Statistics

Descriptive statistics has been performed for the NI and I-group, respectively, regarding the frequency (n) as to age, median, and gender of the patients. The physiological signals for heart rate, galvanic skin response and motion were analysed. The physiological signals for fear and pain at the beginning and end of each intervention were evaluated in relation to the equivalent analog protocol items. The hand movements were analysed correspondingly.

## Results

### Patient groups

The NI-group consisted of 20 patients, 10 girls and 10 boys (14.6 ± 0.5 years), which underwent oral check-ups (Table 1).

**Table 1:** Distribution of patients (*n*) in the dental procedure group (NI) and (I), respectively, gender (*n*) *F* female/*M* male, age median, and number of interventions

Group/procedure	Patients (*n*)	Gender F/M (*n*)	Age median	Interventions (*n*)
Non-invasive (NI)/oral check-up	20	10/10	15.0	20
Invasive (I)/extraction	14	10/4	15.3	27
Total	34	20/14	15.2	47

The I-group consisted of 14 patients, 10 girls and 4 boys (15.3 ± 0.5 years), which underwent permanent premolar extractions (Table 1). The number of the extracted teeth was 27. The distribution of the extracted teeth per patient was as follows: five patients; 1 tooth, four patients; 2 teeth, two patients; 3 teeth, two patients; 4 teeth.

All 34 patients accepted and tolerated the Shimmer3 GSR + device well, without reporting being frightened by it.

### Analogue data of the protocols

Patient mood, as reported by facial analogue scale, ranged between *happy* and being *neutral* for all protocol items during all dental sessions of the NI and I-group. Thus, no patient reported emotional distress.

Fear was considerably more often reported by the I-group than by the NI-group. As reported for each protocol item in both groups, fear showed overall low gradings, 0–2/5.

Pain reports were infrequent in both the I-group and NI-group. In the NI-group, pain was reported in 3/20 interventions, all occurring during the oral radiograph procedure.

Pain intensity, as measured by the CAS for each protocol item in both the I and NI-groups, showed overall ratings < 3.5/10.

### Digital data

The heart rate reading, based on photoplethysmography (PPG), was sensitive to the adjustment of the corresponding finger electrode. In numeral cases, the optical probe was misplaced, resulting in generally uninterpretable PPG reading. In total, PPG data could be read in 5 cases, of which increasing heart rate was noted in 3 cases linked to the injection procedure, I-group.

The hand movements sensor data, as captured by the motion sensors (accelerometer, gyroscope, magnetometer), was interpreted as being adequate. In all I and NI patients, through all procedures, and at some time, hand movements were noted. Most frequently, these were noted during the application of the topical anaesthesia, a non-invasive procedure per se, of the I-group. The hand movements were equally frequent, during the injection performance.

**Fig. 2:**
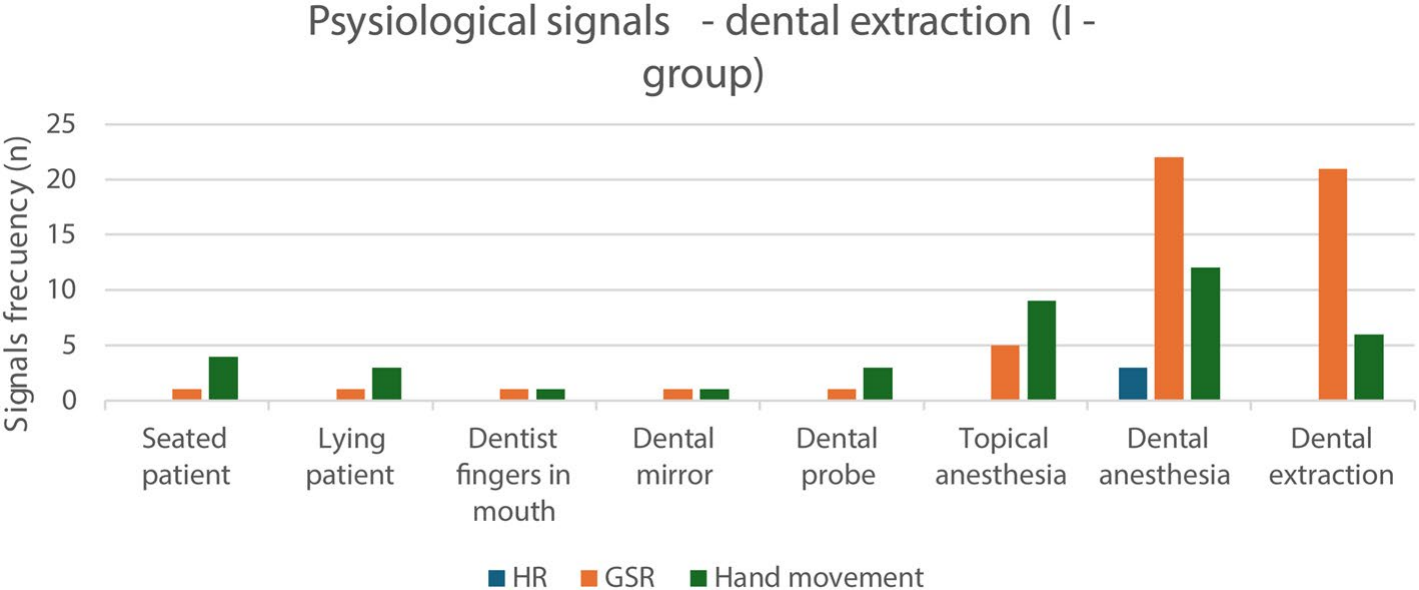
Frequency of physiological signals (*n*) heart rate (HR), galvanic skin response (GSR), and hand movement during the performance of the items included in the dental extraction protocol (I-group)

The galvanic skin response (GSR) data were interpreted as being adequate (Fig. 2). In two I-group patients, the digital readings could not be detected due to technical problems.

GSR ascended substantially in the I-group, at start and during 21 of the 27 injection procedures, and in 3 of the 27 extraction/luxation procedures (Fig. 2). The physiological signals for the tooth extractions followed the same pattern as the tooth luxation.

Patient compliance, as rated by the dentist: All patients of the I and NI-groups cooperated fully, grade 3, to all protocol items.

## Discussion

The main result indicated a significant increase in the galvanic skin responses (GSR), at the starting point and during the oral injection procedure, in the I-group. The GSR amplitudes persisted throughout and post the injection performance. The validity of the GSR readings for the I-group-data was reinforced by the corresponding analogue records, indicating the start of the oral injection. Accordingly, previous reports, among children and adolescents, have found injection to be the most fear and pain provoking dental procedure (Krekmanova and Robertson 2020). Moreover, most of the I-group patients consistently reported mild anxiety specifically connected to the protocol items, injection, dental luxation, and extraction, which the GSR signals supported. Because of the highest GSR signals during the injection, these reactions are interpreted as stress signs, most possibly induced by pain sensations.

In contrast, for the NI protocol items as, using the dental mirror, or the dental probe, and dental radiograph no similar uniform pattern or high GSR amplitudes were produced. Among all detectable physiological stress responses such as heart rate, dilated pupils, and plasma cortisol levels, GSR may possibly be the most easily measurable sign (Rathmell and Fields 2012; Loscalzo et al. 2012; Yang 2011). Though, the possibility of vast interindividual range of physiological responses should be considered. In the NI-group, there was no unanimous high GSR response for specific items which point to overall different physiological reactions in comparison to the I-group.

The fact that no patient, in either group, reported severe fear or pain could be the result of the small study population, as no of the invited patients declined participation. Another explanation could be that the dentist applied iatrosedation and thus influenced the patients’ experience in a positive way (Friedman 1983).

A limitation of the pilot study was that the number of I-group participants did not match the NI-group. Therefore, nine of the I-group patients had more than one tooth extracted which could have influenced the physiological response during the succeeding visit. Consistently, Versloot et al. 2008 found that the pain that children perceived at the second dental injection was strongly influenced by the level of dental anxiety experienced at the first anaesthesia occasion (Versloot et al. 2008).

Another limitation was that the pulse electrode could easily be misplaced on the finger, giving inadequate responses. Despite well-thought-out protocols, the Shimmer3 GSR + unit was applied after the patient was seated for 5 min to calm down, establishing only an analogue baseline.

To our knowledge, there are no studies where patient physiological data have been retrieved with a digital device as in this pilot, recording invasive versus non-invasive dental procedures. Going forward, it is important to find a digital non-invasive method to objectively detect stress in young dental patients and prevent fear development. Most significant is that the method should be easily accepted by the young patients.

An advantage in this study was that no patient discontinued the participation. A further advantage was that the applied device was well accepted by all participants.

In the future, interdisciplinary research is essential for identifying optimal prevention of acute procedural pain in young dental patients. The Shimmer3 GSR + unit or a similar wearable sensor device could potentially provide a future solution.

## Conclusion

Considering the limitations of this study, the following conclusions can be made: the invasive treatment resulted in a specific unison GSR pattern, while the non-invasive procedure showed individually scattered GSR reactions.

The commercially available CE-marked Shimmer3 GSR + device indicated the patient’s stress response triggered by the invasive anaesthetic procedure.

## Data Availability

No new data were created.
